# Role of EGF/ERBB1 in the transcriptional regulation of the prolactin receptor independent of estrogen and prolactin in breast cancer cells

**DOI:** 10.18632/oncotarget.11579

**Published:** 2016-08-24

**Authors:** Raghuveer Kavarthapu, Maria L. Dufau

**Affiliations:** ^1^ Section on Molecular Endocrinology, Eunice Kennedy Shriver National Institute for Child Health and Human Development, National Institutes of Health, Bethesda, MD, USA

**Keywords:** prolactin receptor, EGFR/ERBB1, EGF, ER, STAT5b

## Abstract

Prolactin receptor (PRLR) and epidermal growth factor receptor (EGFR/ERBB1) have important roles in the physiology of the human breast and in the etiology and progression of breast cancer. Our present studies in MCF-7 cells revealed that EGF induces up-regulation of PRLR via activation of EGFR signalling pathways leading to activation of estrogen receptor α (ERα). EGF treatment of MCF-7 cells cultured in absence of estradiol induced expression of PRLR that was consistent with the activation of PRLR generic promoter (hPIII). These were abolished by ERα antagonist and siRNA, indicating involvement of ERα in EGF-induced hPIII promoter activity. MEK/MAPK and PI3K/AKT pathways participate in the phosphorylation of ERα induced by EGF/EGFR. PI3K and MEK inhibitors abolished EGF-induced PRLR promoter activity. Increased recruitment of non-DNA bound unliganded ERα to Sp1 and C/EBPβ bound to their sites at hPIII induced by EGF was abrogated by ERα siRNA demonstrating the requisite role of phospho-ERα in PRLR upregulation. EGF/EGFR, independent of endogenous prolactin induced phosphorylation of STAT5b with participation of c-SRC and recruitment of STAT5b:STAT5b to a GAS site at hPIII. STAT5b interaction with ERα was essential for stable phospho-ERα recruitment to the SP1/CEBPβ complex. These studies indicate a role for paracrine EGF via EGFR independent of estrogen and prolactin in the transcriptional activation of PRLR gene expression and its contribution to high levels of PRLRs in breast cancer. These by maximizing the actions of endogenous prolactin could have a role in cancer progression and resistance to endocrine therapy.

## INTRODUCTION

The long form of the human Prolactin Receptor (PRLR) of 622 amino acids with extended cytoplasmic domain present in the cell membrane of normal and cancer cells mediates all the known diverse functions of prolactin (PRL). Binding of PRL to its ligand site at the most proximal extracellular D1 domain induces conformational changes on constitutive receptor dimers that result in the activation of the JAK2/STAT5 pathway to induce the expression of PRL responsive genes [1–3]. Also, PRL via PRLR induces extracellular signal-regulated kinase 1/2 (ERK1/2) independently of STAT5 with participation of JAK2/c-SRC family kinases/focal adhesion kinase via phosphatidylinositol 3-kinase (PI3K) [4, 5]. In addition of the known primary physiological role of PRLR activation by its cognate hormone in growth and differentiation of mammary gland and lactation, it mediates the development and progression of breast cancer and causes chemoresistance [6]. Short forms of the PRLR S1a and S1b with abbreviated cytoplasmic domain generated by alternative splicing were also found in normal, breast cancer tissues and cells. PRL binds these short forms as homodimers and induces JAK2 phosphorylation while STAT5 activation is absent due to lack of downstream cytoplasmic sequences. These species are inhibitory of the action of PRL long form of the receptor. These result from heterodimerization with the long form and the formation of imperfect dimers which prevents STAT5 signaling. Low ratios of short forms to the long-form of the PRLR are associated with mammary carcinoma [7, 8]. Increased levels of PRLR in breast cancer indicate their participation in the proliferation of breast tumor and cancer cells induced by prolactin [9, 10].

In recent studies, we have demonstrated in breast cancer ERα^+^ and HER2^+^ cells, up-regulation of PRLR transcription/expression induced by endogenous/exogenous PRL in the absence of estrogen via the long form of the prolactin receptor with essential participation of ERα and JAK2/STAT5, mitogen-activated protein kinase (MAPK) and PI3K pathways [5]. In these studies we also found ERBB2/HER2, a member of epidermal growth factor receptor family which is overexpressed in about 10% of the ER^+^ breast cancers [11, 12] phosphorylated and activated by JAK2 induced by PRL through PRLR. Such cross-talk activation of ERBB2/HER2 signalling was identified as an alternate route in the upregulation of PRLR induced by its cognate hormone in breast cancer cells [5].

In the tumor microenvironment various stromal cell types exist in symbiosis within the breast tissue where reciprocal paracrine inputs have an active role in tumor development, progression and metastasis via cross-talk activation of signaling pathways [13–15]. Stromal fibroblasts secrete various paracrine growth factors like EGF, FGF2 and TGFβ that can affect cell proliferation and survival [16]. Epidermal growth factor (EGF) is well known to stimulate proliferation of human breast cancer cells in cultures [17]. The affinity of the receptors for EGF is much higher in ER-positive breast cancer cell lines [18]. EGF through EGFR/ERBB1 activates various downstream signaling pathways which in turn trigger other transcription factors and co-activators that can affect the proliferation of tumor cells. Clinical studies have indicated an increased expression of Epidermal Growth Factor Receptor (EGFR/ERRB1) and its family member ERBB2 in breast cancers which are associated to tumor development, progression [19, 20]. PRLR and EGFR both have important roles in human breast cancer. Most breast cancers that become resistant to endocrine therapy have an increased expression of EGFR with activation of downstream signaling pathways [21, 22]. Since these have commonalities with those involved in the up-regulation of PRLR induced by PRL it was of interest to investigate whether EGFR, a member of the epidermal growth factor tyrosine kinase receptor family has a role in the PRLR gene transcription and expression upon activation with EGF in breast cancer cells.

In the present studies utilizing MCF-7 (ERα^+^ HER2^+^) breast cancer cells which express EGFR/ERRB1 but not its ligand we have shown marked activation PRLR gene at the transcriptional level by exogenous EGF with the essential involvement of the mitogen-activated protein kinase (MAPK; ERK1/2) and PI3K-AKT signaling pathways mediated through activation of EGFR. Moreover, c-SRC dependent EGFR-mediated receptor events was also established. The PRLR regulation by EGF was independent of PRL/PRLR/JAK2 induced regulation previously reported [5]. Further, activation of downstream effector genes induced by EGF/EGFR signaling are essential for up-regulation of PRLR transcription by EGF/EGFR. These findings provide a novel additional mechanistic avenue for the increase of PRLR in cancer to maximize the actions of endogenous/exogenous PRL that upon resistance to hormonal therapy could promote progression and metastasis in breast cancer.

## RESULTS

### Effect of EGF on hPRLR transcription/expression

**Figure 1:**
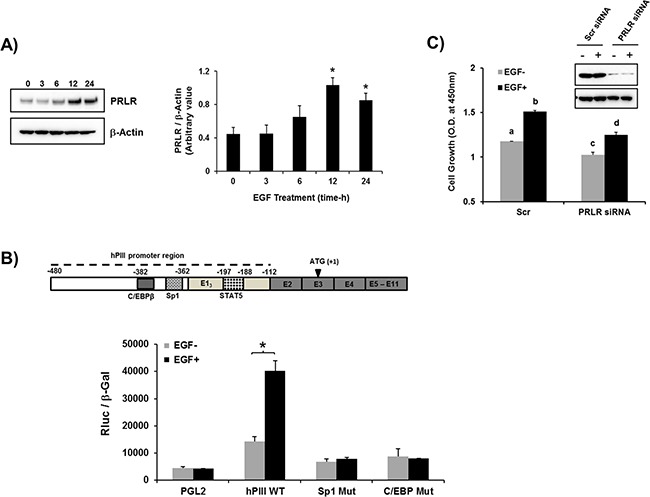
EGF induced up-regulation of PRLR gene transcription/expression **A.** Time course of EGF stimulation of the long-form PRLR protein in MCF-7 cells. Western blots detected by PRLR antibody (left). Graph showing relative PRLR protein levels were normalized by endogenous β actin levels (right). Asterisks (*) indicate Statistically significant increase in PRLR protein levels in 12 h and 24 h times when compared to 0 h time point (Tukey's multiple comparasion test; *P* < 0.01). **B.** Effect of EGF (100 ng/ml for 16 h) on PRLR promoter activity of cells transfected with PGL2 construct (control) or wild type hPIII/hE1_3_ (-480/-112, includes promoter and non-coding exon 1 which is require for promoter activity [26] or hPIII constructs with Sp1 and C/EBPβ functional DNA binding sites at the promoter mutated. Results presented are relative luciferase activities (Rluc) normalized to the activities of co-transfected β-galactosidase. Asterisks (*) indicate Statistically significant changes between EGF untreated and treated groups (Student *t*-test; *P* < 0.05 Results in these and in Figures below are reported as the mean ± SE of three independent experiments. **C.** Evaluation of PRLR on MCF7 cell proliferation induced by EGF in controls and PRLR knock-down cells by Scrambled (Scr) and PRLR siRNA, respectively following stimulation by EGF (100 ng/ml) for four days (see materials and methods section). Western blot of PRLR knockdown. Lower case letters indicate groups evaluated by Tukey's multiple comparasion test as follow: a versus b (*P* < 0.001); c versus d (*P* < 0.01); a versus c (*P* < 0.05); b versus d (*P* < 0.01).

Initially we evaluated whether EGF induces PRLR gene expression and transcription in MCF-7 cancer cells. In these cells, which are known to possess EGFR but lack the endogenous cognate hormone, addition of EGF caused a significant upregulation PRLR protein expression that reach a peak levels at 12 h and remained elevated at 24h (Figure 1A). We observed an increase in PRLR promoter activity (3-fold) in cells transfected with the wild type hPIII promoter fused to reporter luciferase. Mutation of SP1 or C/EBPβ sites at hPIII prevented stimulation of promoter activity by EGF displaying levels comparable to control mutants which were reduced when compared to basal wild type controls (Figure 1B; above hPIII PRLR promoter). Thus, the stimulation of PRLR expression resulted from activation events at the promoter with participation of transcription factors Sp1 and C/EBP which are known to participate in other PRLR gene regulatory events induced by hormones including estradiol (E2) and prolactin per se in absence of estradiol [5, 23, 24]. EGF treatment significantly stimulated cell growth in MCF-7 cells transfected with scramble siRNA (control), and knock-down of PRLR in MCF-7 cells significantly reduced basal and EGF induced cell growth compared to controls. These studies indicated contribution of PRLR and EGF/ERBB1 in cell proliferation (Figure 1C).

### Role of ERα and STAT5 in EGF induced promoter activity

**Figure 2:**
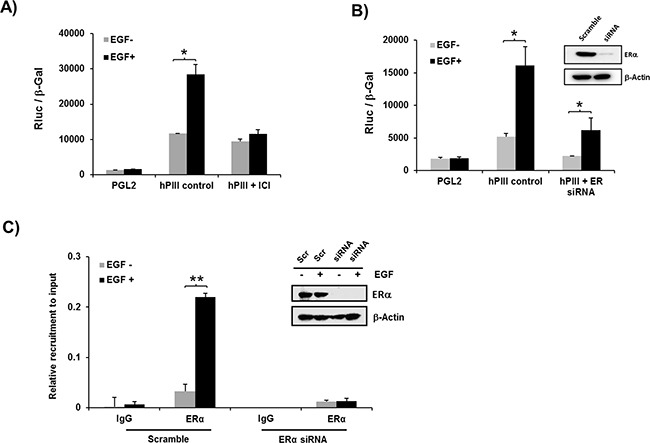
Role of ERα on EGF induced promoter activity A, B. and recruitment of ERα to the PRLR promoter C **A.** Effect of EGF on PRLR promoter activity of cells transfected with pGL2 vector (basal) or hPIII construct in presence or absence of ERα antagonist, ICI 182,780 for 24 h (left) or **B.** transfected with coding region of ERα siRNA or scramble (Scr) siRNA (control) Inset, shows Western blot of ERα knockdown. Asterisks (*) indicate Statistically significant changes between EGF untreated and treated groups (Student *t*-test; *P* < 0.01). **C.** Chip assay showing recruitment of endogenous ERα in cells transiently transfected with coding region of ERα siRNA or Scramble siRNA (Inset, Western blot of siRNA knockdown). Asterisks (**) indicate Statistically significant changes between EGF untreated and treated groups (Student *t*-test; *P* < 0.001).

The activation of PRLR hPIII promoter by EGF was completely prevented when cells were pre- incubated with the ERα antagonist ICI which promotes receptor degradation (Figure 2A). Moreover, transfection of cells with ERα siRNA with effective depletion of the nuclear receptor prior to EGF addition to the cultures, showed a significant reduction of basal to empty control vector (PGL2) levels and of EGF stimulated activity to levels comparable to basal controls (Figure 2B). In ChIP assays, EGF stimulation of cells transfected with scramble siRNA showed significant increased ERα recruitment to the PRLR promoter when compared to untreated control. In contrast, the observed recruitment to the EGF stimulus was abolished in cells transfected with ERα siRNA which effectively reduced the endogenous levels of ERα (Figure 2C). Taken together these findings demonstrate the relevance of ERα in absence of estradiol on EGF induced up-regulation of PRLR gene activation.

**Figure 3:**
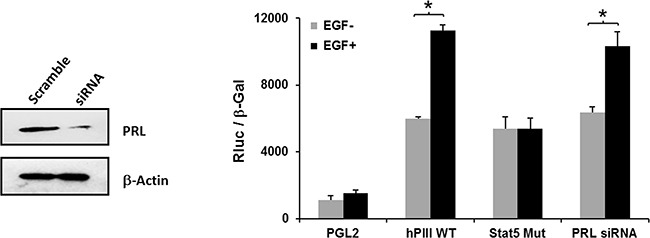
Relevance of STAT5 and lack of participation of endogenous PRL on EGF activation of PRLR promoter activity Cells transfected with PGL2 or PIII WT or PIII with STAT5 site mutated construct a group cells were co-transfected with PRL siRNA for 24 h and subsequently incubated with EGF or buffer control. Depletion of endogenous PRL by siRNA versus scramble siRNA (Scr) are shown in the Western blot (left). Asterisks (*) indicate Statistically significant changes between EGF untreated and treated groups (Student *t*-test; *P* < 0.05).

The hPIII PRLR promoter contains proximally in non-coding exon 1, a functional STAT5 response element (-197/-188) that was found to bind STAT5a and STAT5b in our previous study [5]. These are required in concert with ERα (non-DNA bound which associates to SP1 and C/EBPβ bound to their cognate DNA sites) for PRLR hPIII activation/and gene expression induced by endogenous/exogenous PRL through its cognate receptor [5]. Mutation of the STAT5 element at the hPIII promoter completely abolished EGF activation of PRLR promoter activity (Figure 3). In contrast, transfection of cells with PRL siRNA that caused significant reduction of endogenous PRL from the cells, caused minor non-significant reduction of EGF stimulation of PRLR hPIII promoter activity. These findings showed a requirement of STAT5 activated by EGF in the up-regulation of the PRLR and indicated that endogenous PRL is not require or contribute to PRLR stimulation of gene transcription/expression by EGF.

### Signal transduction pathways induced by EGF that participate in ERα phosphorylation

**Figure 4:**
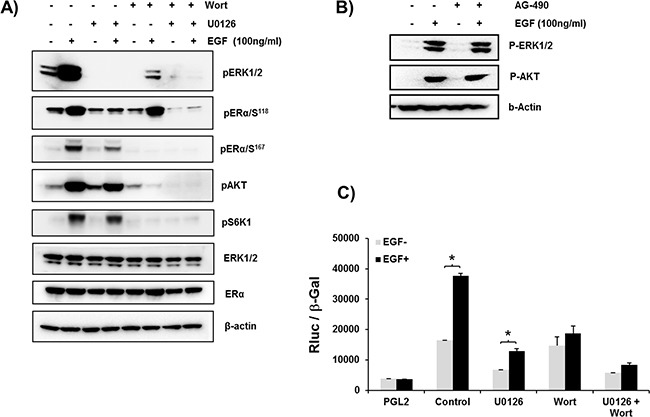
**A. ERα phosphorylation induced by EGF through the PI3K/MAPK kinase pathway.** Representative Western blot showing the phosphorylation status of ERα (ser118 and ser 167), ERK1/2, pAKT and pS6K1 in MCF-7 cells in controls and after treatment for 30 min with EGF. Endogenous ERK1/2, ERα and Δ-actin are the loading controls. The above phosphorylation Status of controls and EGF-induced parameters were also assessed following pretreatment of cells with MEK in inhibitor, U0126 (10μM) or Wortmannin (0.5 μM)(Wort) or both inhibitors for 2 h prior to the addition of EGF. **B. Lack of participation of JAK2 in MAPK and PI3K pathways induced by EGF.** Western blot showing pER1/2, and pAKT phosphorylation of cells cultured in presence or absence of EGF for 1 h following preincubation with or without JAK2 inhibitor AG-490 for 2 h. Δ actin was used as loading control. **C. EGF-induced up-regulation of hPIII transcriptional activity abolished by ERK1/2 and PI3K inhibitors.** Cells transfected with hPIII promoter construct or PGL2 vector (control) were pre-incubated with inhibitors U0126 or Wortmanin (Wort) for 2 h prior to addition of buffer in controls or the EGF stimulus and further incubated for 16 h for evaluation of promoter activity. Asterisks (*) indicate Statistically significant changes between EGF untreated and treated groups (Student *t*-test; *P* < 0.05).

EGF treatment of cells caused phosphorylation of ERα at S^167^ by MEK/ERK and PI3K pathways and of S^118^ by MEK/ERK (Figure 4A). Major inhibition of phosphorylation induced by EGF was observed for both pERα/S^118^ and pERα/S^167^ upon incubation with inhibitor of MEK/ERK pathway which completely abolished pERK1/2 phosphorylation/activation. In contrast, Wortmanin which effectively inhibited the activation of the PI3K pathway, as shown by abolition of pAKT/S6K1 phosphorylation, prevented the phosphorylation of ERα at S^167^ while causing minor or no change on the EGF-induced phosphorylation of ERα at S^118^ despite the reduction of EGF-induced pERK1/2 by Wortmanin. Wortmanin is also known to attenuate phosphorylation of ERK1/2 as it inhibits PH-domain containing adaptor protein Grb2-associated binder 1 (GAB1) an additional positive regulator of ERK that lay upstream of MEK. It also inhibits PI3K-activated PAK which in turn can phosphorylate ERK1/2 [27, 28]. The addition of both inhibitors caused abolition of ERK and pAKT/pS6K1 phosphorylation resulting in complete abolition of ERα at Ser 118 and 167 (Figure 4A). These results indicated the participation of both signal transduction pathways in EGF induced phosphorylation of ERα. Moreover, phosphorylation of ERK 1/2 and AKT induced by EGF was not affected by pre-incubation of cells with the inhibitor of JAK2 kinase AG-490 (Figure 4B) demonstrating the lack of participation of JAK2, constituitive or endogenously induced, in the EGF signaling activation. The relevance of these findings was indicated by the major inhibition of EGF induced hPIII promoter activity by MEK/ERK or PI3K inhibitors. Pre-treatment of cultures with MEK/ERK1/2 inhibitor significant reduced basal promoter activity to below basal control and similarly marked reduction to 50% of EGF stimulated levels was observed. In contrast, basal promoter activity was unchanged by the PI3K inhibitor while EGF stimulated levels were reduced to basal levels (Figure 4C). The lack of change in basal could be attributed to a contribution of the MEK/ERK1/2 presumably independent of PI3K supported by endogenous prolactin action in its cognate receptor and indicated by the presence of pERK1/2 in the cultures treated with wortmanin and EGF (Figure 4A). However, because wortmanin completely inhibited basal pERK1/2 levels this lack of basal inhibition would require further investigation. The complete inhibition near basal control (PGL2) levels caused by the addition of both inhibitors in control and EGF-simulation underscored the relevance of MEK/ERK1/2 and PI3K in the transcriptional control of the PRLR (Figure 4C).

### STAT5b phosphorylation by EGF/EGFR via c-SRC and its interaction with ERα

**Figure 5:**
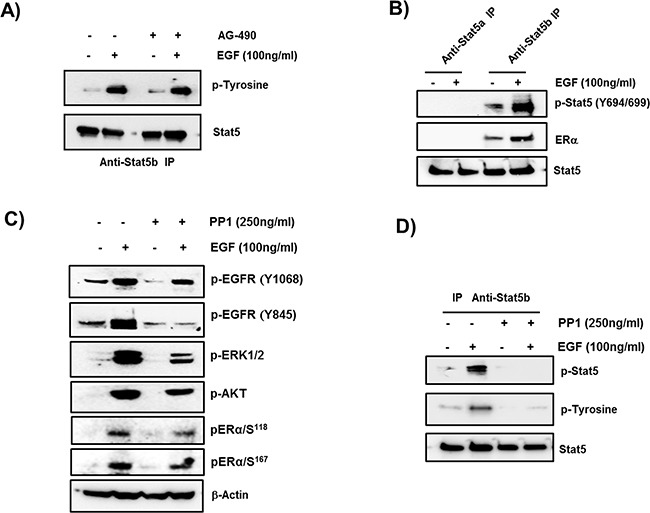
Role of STAT5b in EGF- upregulation of PRLR **A. Phosphorylation of STAT5b by EGF.** Western blots of extracts from cells incubated in presence of absence of EGF for 1 hour following preincubation with or without JAK2 inhibitor AG-490 for 2 h, immunoprecipitated with STAT5b antibody and probed with anti-phospho Tyrosine antiserum. STAT5b was used as loading control. **B. Phosphorylation of STAT5b and its interaction with ERα.** Western blots of STAT5a or STAT5b immunoprecipitates of extracts from cells incubated with or without EGF that were probed with pSTAT5 (Y694/699) or ERα. The presence of ERα in the immunoprecipitated samples indicate its interaction with STAT5b. STAT 5 is the loading control, **C. Evaluation of EGF activation in presence and absence of c-SRC inhibitor PP1.** Western blots of EGF dependent phosphorylation of EGFR at p-EGFR at Y1068 and Y845. pERK1/2 and pAKT indicative of downstream pathways activation by EGFR kinase and pERα at Ser 118 and 167. β-actin used as internal control. **D. Effect of PP1 on EGF induced phosphorylation of STAT5b.** Western blots of extracts cells incubated with EGF or buffer control following pre-incubation with or without 250 ng PP1 for 2 h. Extracts immunoprecipitated with Anti-STAT5b antibody were probed by pSTAT5 and p-Tyrosine antibodies. STAT5 was used as internal control.

Immuno-precipitates from protein extracts of MCF-7 cells stimulated with EGF by STAT5b antibodies, revealed tyrosine-induced phosphorylation in Western blots upon exposure to Tyr-phosphorylated antibody which was not affected by AG-490 antagonist (Figure 5A). To determine the STAT5 class induced by EGF/EGFR responsible for up-regulation of the PRLR cell extracts control and stimulated by EGF were immunoprecipitated by STAT5a or STAT5b specific antibodies and phosphorylation of individual STAT5 was monitored by exposure of Western Blots to anti phospho pSTAT5 (Y694/699). Only STAT5b was found to be phosphorylated upon EGF/EGFR activation of the cells. Moreover, association of ERα with pSTAT5b was revealed in this study (Figure 5B). EGF induced phosphorylation of EGFR at Tyr residue 845 was higly reduced by c-SRC inhibitor PP1. While PP1 at 250 nM concentration minimaly affected the EGF induced auto-phosphorylation of EGFR at Tyr 1068 residue (Figure 5C). This low dose of the antagonist permitted to dissect c-SRC/EGF/EGFR mediated signaling actions impacting Stat5b phosphorylation (EGFRphospho-Y845) from those of EGF/EGFR kinase (EGFR phospho-Y1068) which activates MEK/ERK and PI3K pathways concerned with phosphorylation of ERα. PP1 cause only minor reduction of EGF induced EGFR phosphorylation at Y1068. However, both MAPK/ERK1/2 and PI3K pathways induced adequate phosphorylation of pERα at S118 and S167 respectively. pERK1/2 and pAKT were reduced but to a degree that preserve adequate ERα phosphorylation (Figure 5C). PP1 used at higher concentration (500 nM) also inhibits EGF induced auto-phosphorylation of EGFR at Tyr 1068 residue (data not shown) and the dissociation of the EGF/EGFR activating pathways could not be studied. STAT5b phosphorylation by EGF/EGFR was abolished by PP1 (Figure 5D) indicating intermediate role of c-SRC in EGFR phosphorylation at Tyr 845.

### EGF induces recruitment of STAT5b onto the hPIII promoter

**Figure 6:**
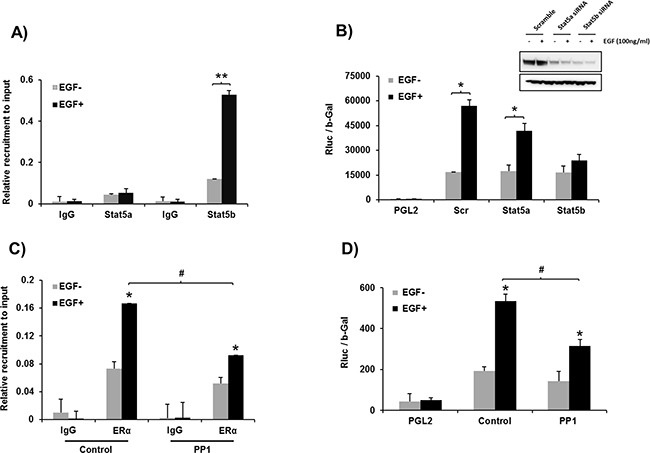
**A. Recruiment of STAT5a and STAT5b onto hPIII promoter.** ChIP assay showing the recruitment STAT5b induced by EGF vs basal. Minor recruitment of STAT5a basal and lack EGF-induced is present. Asterisks (**) indicate statistically highly significant increase in recruitment between EGF untreated and treated groups (Student *t*-test; *P* < 0.001). **B. Requisite role of STAT5b in PRLR transcription.** Promoter activity of cells depleted of STAT5a or STAT5b by specific siRNAs of scrambled transfected PGL2 and hPIII constructs and incubated with EGF or buffer control. Asterisks (*) indicate Statistically significant increase in recruitment between EGF untreated and treated cultures (Student *t*-test; *P* < 0.05). Western blot (Inset) show the efficacy of siRNA knockdown. **C. Effect of PP1 on ERα recruitment to the hPIII promoter.** PP1 which significantly reduces EGF/EGFR induced recruitment of ERα (Tukey's multiple comparision test; #, *P* < 0.01), **D. Effect of PP1 on EGF induced PRLR promoter activity.** Significant inhibition of EGF induced PRLR promoter activity was observed in PP1 treated group compared to control group (Tukey's multiple comparision test; #, *P* < 0.01).

EGF induced recruitment of STAT5b onto the hPIII promoter where a 5-fold increase from basal levels was observed. Only a minor basal recruitment of STAT5a was present with levels below those of basal for STAT5b and no significant changes in recruitment were induced by EGF (Figure 6A). Moreover, knockdown of STAT5b, abrograted hPIII promoter activity induced by EGF, in contrast significant activation was observed when STAT5a was effectively reduced by siRNA (Figure 6B). c-SRC inhibitor significantly reduced the EGF induced recruitment of ERα onto the PRLR promoter (Figure 6C). We also observed a decrease in the hPIII promoter activity induced by EGF in cell treated with c-SRC inhibitor compared to control cells without any treatment (Figure 6D). Taken together these results indicate that both ERα and STAT5b are required for the up-regulation of PRLR by EGF and the participation of c-SRC in this process. Selective inhibition of c-SRC by low dose PP1 prevents phosphorylation of EGFR at Y845, STAT5b and its recruitment to the DNA site at the hPIII. In contrast ERα phosphorylation induced by EGF is maintained via EGFR kinase (pEGFR-Y1068) whose activation is independent of c-SRC through MEK/ERK and PI3K/AKT/TOR/S6K1 pathway.

## DISCUSSION

Our studies have demonstrated that EGF activation of EGFR through the intrinsic tyrosine kinase activity of the receptor and the activation of downstream signal transduction pathways (MAPK/ERK and PI3K/AKT), up-regulates the human prolactin receptor. Moreover, c-SRC dependent EGF/EGFR induced events participate in this regulation. The essential role of both pERα and pSTAT5b in EGF/EGFR induced activation of PRLR transcription was also established in this study. ERα phosphorylation is induced by EGF/EGFR via the MAPK/ERK and PI3K/AKT pathways without the participation of JAK2. EGF via EGFR kinase activity phosphorylates STAT5b, and requires participation of c-SRC which phosphorylates EGFR at Y845. IP analysis has revealed interaction between ERα and STAT5. Chip studies demonstrated that pSTAT5b bound at its DNA site interacts with pERα which forms a complex to Sp1 and C/EBPΔ dimers bound to their respective sites at the hPIII promoter. A functional GAS element which binds STAT5 was previously identified in the non-coding exon 1 of the PRLR [5]. These findings provide mechanistic insights whereby EGF, through its EGFR and the requisite participation of the STAT5b and ERα, promotes the up-regulation of PRLR in the absence of estrogen. In addition, a contribution of PRL/PRLR and EGF/ERBB1 was observed in the proliferation cells basally and following stimulation with EGF. Prolactin and EGF and their receptors were found to be associated with breast cancer progression and to enhanced MCF7 and T47D cell proliferation [29, 30]. EGF in this study was shown to increase PRLR transcription and expression. Thus, it is conceivable that some of EGF actions could result from the activation of PRLR by endogenous prolactin expressed in tumor cells.

EGF through EGFR/ERBB1 activates various downstream signaling pathways which in turn trigger other transcription factors and co-activators that can affect the proliferation of tumor cells. In our studies we have shown that EGF induces phosphorylation of ERα and STAT5b and their recruitment to the hPIII promoter. Phosphorylation of ERα induced by EGF/EGFR which is a requisite for PRLR promoter up-regulation was abolished by pre-incubation of cells with inhibitors of MEK and MAPK signaling pathways. Previously we reported that ERα through complex formation with C/EBPβ and Sp1 increased transcription/expression of PRLR over basal constitutive levels observed in the absence of E_2_. ERα exists as constitutive homodimers in the absence of hormone and E_2_ favors its association with homodimers of Sp1 and C/EBPβ. *In vivo* approaches determined the DNA binding domains of ERα-dimer as specific sites of interaction with the 2^nd^ and 3^rd^ Zinc Fingers (ZF) of SP1-dimer and the leucine zipper of C/EBPβ-dimer within the complex [24]. Moreover, the basic region of C/EBPβ in addition to the LZ were require for interaction with ZPs of Sp1 and stabilization of the complex [24]. It is also indicated that ERα is initially recruited to Sp1 bound to its site and this is follow by the recruitment of C/EBPβ to the complex. The interaction of ERα to the recruited STAT5 dimer at its downstream element are required for the stable association of ERα to the complex and up-regulation of receptor transcription/expression [5]. Thus, whether up-regulation of the PRLR is induced by PRL/PRLR or EGF/EGFR the association of ERα to the STAT5 a & b or STATb, respectively are/is required for recruitment of ERα to form the Sp1/C/EBPβ complex essential for activation of transcription/expression of PRLR gene.

To date we have found three modalities for PRLR up-regulation at the transcriptional level with consequent increases in the expression of a functional PRLR in breast cancer cells. These include in addition to the positive regulation induced by estrogen through activation of ERα reported in our early study [23, 24] two estrogen-independent ERα inductive mechanisms – the recently described regulation of PRLR by its cognate hormone [5] and in the present study by EGF/EGFR. The latter was shown to be independent of JAK2 and therefore of PRL/PRLR/JAK2 participation. Moreover, aside from its lack of dependence of E2 and the requirement of different activators (PRL/PRLR and EGF/EGFR) there are important commonalities in the mechanism for PRLR transcriptional activation/expression.

Previously we demonstrated binding of both of STAT5 to a functional GAS site in non-coding exon-1 of PRLR induced by PRL/PRLR, and the recruitment of pERα to Sp1/C/EBPβ dimers bound to their sites at the promoter. We also established the requirement of binding of both STAT5a & b variants to the DNA site and proposed their participation as heterodimers and/or tetramers formed from homodimers. In contrast, in this study we have shown that STAT5b not STAT5a is required for EGF induced hPIII promoter activity. Further, we have shown that phosphorylation of STAT5b induced by EGF/EGFR is required for its recruitment to PIII promoter. In addition our studies revealed STAT5b interaction with ERα which is also recruited to the hPIII promoter and is essential for the PRLR gene activation. Furthermore, we determined by ChIP/reChIP association of STAT5 with ERα bound to the Sp1/C/EBPβ complex upon PRL stimulation [5]. The present evidence indicates that unliganded ERα is stabilized by its association with STAT5 bound at its site in the promoter. Previous studies by others demonstrated cross-talk between STAT5b and ERα where ERα supported transcription of β-casein STAT5 dependent promoter activation by PRL in COS-7 cells and the requirement of the DNA-binding/hinge domain for this activation [25].

**Figure 7:**
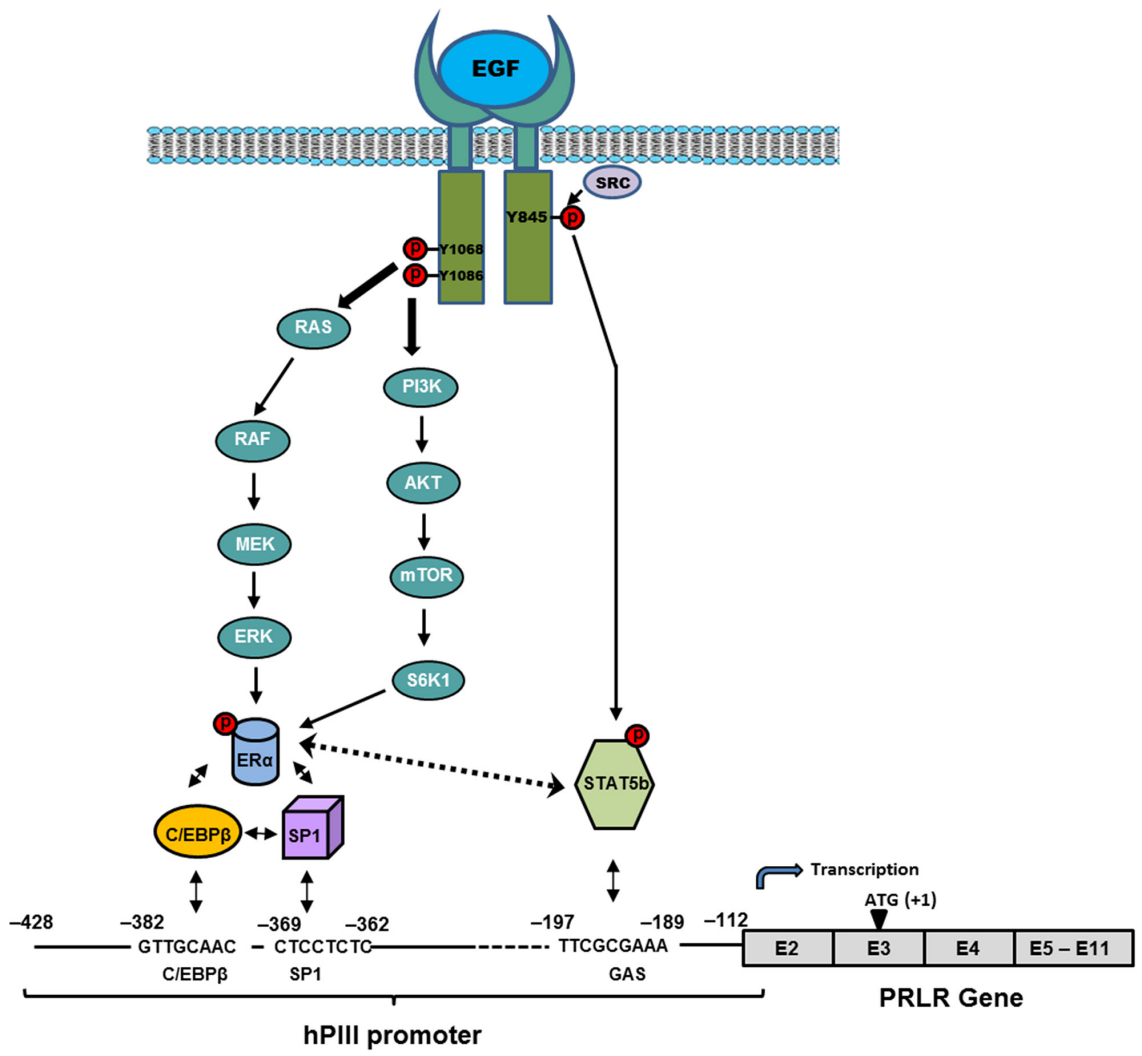
Proposed mechanism of up-regulation of the PRLR by EGF/EGFR Signal transduction mechanisms induced by EGF via EGFR causing activation of transcription factors which are require for PRLR gene activation through the hPIII promoter and consequent increased expression of the receptor in MCF-7 cells.

Taken together, our studies have demonstrated essential role of ERα and STAT5b in EGF/EGFR induced activation of PRLR gene transcription/expression in breast cancer cells via STAT5b interaction with ERα and complex formation with Sp1/C/EBPβ at the PRLR promoter in the absence of estrogen. The participation of the MAPK/ERK and PI3K/AKT pathways is required for phosphorylation of ERα and of c-SRC/EGFRY845 in STAT5b phosphorylation for their recruitment to the PRLR promoter (Figure 7). These studies providing mechanistic insights into the up-regulation of the PRLR by EGF hormone and its receptor kinase indicate the relevance of their joint participation in tumor progression and resistance to adjuvant therapies and further the basis for the treatment of refractory states in ERα^+^ breast cancers.

## MATERIALS AND METHODS

### Reagents and antibodies

RPMI 1640-GlutaMAX and Phenol-red free RPMI 1640 media were obtained from ThermoFisher Scientific. Charcoal stripped fetal bovine serum was purchased from Atlanta Biologicals. ERα, STAT5a, STAT5b, STAT5, β-actin antibodies and inhibitors of PI3K (Wortmannin) and JAK2 (AG-490) and c-SRC (PP1) were obtained from Santa Cruz Biotechnology (Santa Cruz, CA). Phospho (p)-ERα (Ser118 and Ser167), pSTAT5, pERK1/2, pMEK1/2, ERK1/2, pAKT, pEGFR (Y1068), pEGFR (Y845) and p-Tyrosine antibodies and U1026 (MEK1/2 inhibitor) were purchased from Cell Signaling. ICI 182,780 (Fulvestrant) and PRLR antibody were obtained from Sigma-Aldrich. Human PRL antibody were obtained from National Hormone and Peptide Program, Harbor-UCLA Med. Ctr., Torrance, CA 90502.

### Cell culture and reporter gene assay

The ER^+^ MCF-7A2 cells (MCF-7) (gift from E. Berleth, C. Roswell Park Cancer Institute, New York, NY) were maintained in RPMI 1640 medium supplemented with 10% charcoal stripped fetal bovine serum at 37°C in CO_2_ incubator. Cells were cultured in 6 or 12 well plates in steroid-free conditions using phenol red-free RPMI media with 5 and 1% charcoal-treated fetal bovine serum for 2 days at each serum concentration. The PRLR generic promoter (hPIII promoter/non-coding exon 1 [hE1_3_]) with reporter pGL2 gene construct (bp −480/−112) containing C/EBPβ, SP1 sites in hPIII and putative STAT5 binding site in hE1_3_, and other constructs with the sites (C/EBPβ, SP1 and STAT5) mutated, and pGL2 empty vector were used for transient transfection in MCF-7 cells using Lipofectamine 2000 reagent (ThermoFisher Scientific) as described previously [23]. Cells were treated with EGF (100 ng/ml) in RPMI phenol red-free media without any FBS for overnight. After the treatment cells harvested to the determine promoter activity using luciferase assay kit (Promega).

### Cell proliferation assay

Cyto Select WST-1 Cell Proliferation Assay (Cell Biolabs) which is based on the formation of formazan in the presence of cellular NADH and an electron mediator was used to asses the effect endogenous PRLR on EGF-induced MCF-7 cell growth. MCF-7 cells (5 × 10^4^ cells/well) were cultured in 24 well plates with phenol red free RPMI media containing 0.5% charcoal stripped fetal bovine serum at 37°C in CO_2_ incubator. The MCF-7 cells were transfected with 10 nM scrambled and PRLR silencer select siRNAs using siPORTNeoFX reagent (Life Technologies) as described previously [24]. Then EGF was added to the media each day to eliminate possible EGF degradation and the cells were cultured for 4 days in charcoal stripped fetal bovine serum. On day 5 WST-1 reagent (50 μl) was added to each well and plates were incubated for 1-2 h at 37°C in CO_2_ incubator until media turns orange in color. Absorbance at 450 nm was read with a Tristar-2 microplate reader (Berthold Technologies).

### Western blot analysis

Whole cell lysates from MCF-7 cells cultured in presence and absence of EGF (100 ng/ml) or treated with either or both U0126 and Wortmannin inhibitors were extracted using RIPA lysis buffer (Thermo Scientific, Rockford, IL) in presence of 1x protease and Phosphatase inhibitor cocktail (Thermo Scientific). The protein samples were resolved on NuPAGE 4 to 12% Bis-Tris gradient gels and transferred to nitrocellulose membranes (ThermoFisher Scientific). Membranes were blocked with 5% skimmed milk powder in phosphate buffer saline and incubated with different primary antibodies and β-actin was used as loading control. Immunodetection was performed using super-signal chemiluminescence system (Pierce).

### siRNA analysis

Silencer Select pre-designed validated siRNAs from Ambion (ThermoFisher Scientific) were used to knock-down the endogenous expression of STAT5a, STAT5b, ERα, PRL, PRLR and scrambled siRNA was used as negative control. These siRNAs with 80-90% knock-down efficiency were validated previously in MCF-7 cells in our laboratory [5]. The MCF-7 cells were transfected with 25 nM siRNA using siPORTNeoFX reagent (Life Technologies) as described previously [24]. After 48 h of siRNA transfection cells were grown in charcoal treated serum 5% for 2 days and 1% for another 2 days. The cells were harvested for Chip assays after treatment with EGF (100 ng/ml) in serum free medium for 16 h. All siRNA sequences are shown in Table 1.

**Table 1:** List of validated siRNAs used for gene knockdown and their sequence information

Gene Name	siRNA Sequence (Sense)
ERα	CAGGCACAUGAGUAACAAATT
STAT5A	AUGGAUAUGUGAAACCACATT
STAT5B	CACCCGCAAUGAUUACAGUTT
PRLR	CCAUGAAUGAUACAACCGUTT
PRL	GCGAAUUCGAUAAACGGUATT
Scramble	AATTCTCCGAACGTGTCACGT

### Chromatin immunoprecipitation (ChIP) assay

ChIP assays were performed using MAGnify^TM^ Chromatin Immunoprecipitation system from Invitrogen according to the manufacturer's protocol as described previously [24]. The relative binding of proteins of interest to the DNA binding sites on PRLR promoter was quantitatively evaluated by real-time PCR assay of the precipitated DNA and input DNA using SYBER Green Master Mix in an ABI 7500 sequence detection system. The primers utilized for amplification of the hPRLR gene promoter sequence that spans the GAS site and Sp1 and C/EBPβ sites are 5′GCATGCTGAAGAAAATCACTGTTTTGCC3′ (forward) and 5′ TGCACGAGGACATGAAGCTCCA 3′ (reverse).

### Statistical analysis

The significance of the differences among groups in EGF induced samples at different time intervals was determined by multiple Tukey's multiple-comparison test (one-way ANOVA) and significance of the differences between EGF untreated and treated groups were determined by Student's *t-*test using the Prism software program (GraphPad Software, Inc, San Diego, California).
